# Creation of a novel imprinting locus

**DOI:** 10.1186/1756-8935-6-S1-P82

**Published:** 2013-03-18

**Authors:** David Taylor, Chelsea Brideau, Warren Wu, Alex Wang, Paul Soloway

**Affiliations:** 1 Genetics, Genomics and Development, Cornell University, Ithaca, NY 14850, USA

## Background

Much work has been done to elucidate the frans-acting proteins that lay down different epigenetic modifications; however, very little is known about the *cis* regulatory regions that direct the modifications to their targets. To investigate this question we use imprinting, parental-specific methylation which results in monoallelic expression, as a model system. The *Rasgrf1* imprinting locus in particular stands out as a tractable system because of the depth of detail to which the DNA methylation mechanism has been described. Previous work in the lab has shown that the *Rasgrf1* gene is regulated by an imprinting control region (ICR) that lies 30kb upstream of the gene’s promoter in between the gene and its enhancer. This ICR consists of a differentially methylated region (DMR) just 5’ of a repetitive element. Data suggest that CTCF binding to enhancer blocking sequences within the DMR is modulated by methylation status, which results in differential interference of enhancer-promoter communication. In somatic tissue the maternal DMR is unmethylated with CTCF bound, disrupting the interaction between the *Rasgrf1* enhancer and promoter and subsequently inhibiting transcriptional activation. The paternal DMR, however, is methylated with no CTCF binding, which allows for the enhancer interaction and consequent transcription. Previous studies have shown the elements which are necessary for imprinting, however, no study has demonstrated what is sufficient for the imprinting phenotype. To test to see whether we understand what is sufficient for imprinting at this locus, we have inserted the *Rasgrf1* ICR between a non-imprinted gene and its enhancer to see if we can create an ectopic imprinting site.

## Materials and methods

Due to its dependence on its well-defined enhancer and haplosuffkiency, the Wnt1 gene was chosen as the target for ICR insertion between its enhancer and promoter. Bisulfite sequencing was used to assess DNA methylation for both germline and somatic tissue. Differential restriction analysis of reverse transcription polymerase chain reaction products was used to determine gene expression from the two alleles.

## Results

We find that somatic methylation on the construct has been laid down as expected: when passed through the maternal germline it is not methylated and when passed through the paternal germline it is fully methylated. Also as expected, the Wnt1 allele under the control of the imprinting locus is expressed during paternal transmission. More characterization must be done to assess the expression of a maternally transmitted allele. Ongoing studies will also investigate the methylation in both the male and female gametes.

## Conclusions

Our lab has successfully genetically reconstituted elements of the ICR. Further investigation will determine if the system fully recapitulates the expected *Rasgrf1* imprinting; however, if it is successful then this system will be the first example of an endogenous gene being modified to become a novel imprinting locus, illustrating that we fully understand what is sufficient for imprinting at the *Rasgrf1* region.

